# Vision Loss Secondary to Meningioma in the Pregnant Patient

**Published:** 2017-09-11

**Authors:** Matthew B Greenberg, Nandini Venkateswaran, Ann Q Tran, Ranya G Habash, Wendy W Lee

**Affiliations:** 1 Department of Ophthalmology, University of Miami Miller School of Medicine, Miami, Florida, USA; 2 Department of Ophthalmology, Bascom Palmer Eye Institute, Miami, Florida, USA

## Abstract

A 31-year-old primagravid female at 27 weeks gestation presented to the emergency room with three weeks of progressive blurring of vision associated with intermittent headaches. Ocular examination revealed diminished visual acuity, decreased color discrimination, and constricted confrontation visual fields; optic nerve appearance was however normal. Magnetic resonance imaging of the brain and orbits revealed a large tuberculum meningioma compressing the optic chiasm and prechiasmatic optic nerves, as well as a small sphenoid wing meningioma. Given the risk of permanent vision loss, the patient underwent emergent tumor resection. Near total resection of the masses was achieved and the patient had complete resolution of her vision post-operatively. She gave birth via Caesarean section at 39 weeks. This case report describes the clinical presentations of intracranial meningiomas and discusses the challenges this condition poses in management during pregnancy.

## Introduction

Meningiomas remain the most common type of primary central nervous system tumor found in adults and can lead to a variety of clinical manifestations depending on factors such as size, location, and extent of brain invasion [1]. Occasionally, these tumors can invade the optic pathway, leading to significant vision loss [2,3]. Hormonal changes associated with pregnancy can lead to growth of meningiomas with consequent visual deterioration, leading to an increased incidence of these rare cases in this population [3]. Thus, meningiomas should be considered as part of the differential diagnosis of the pregnant patient with worsening vision loss. Herein, we present a unique case of a pregnant woman with progressive vision loss secondary to suprasellar and sphenoid wing meningiomas who underwent surgical resection of masses prior to delivery, with restoration of vision. We use this case to describe the clinical presentations of meningiomas and discuss the challenges this condition poses in management during pregnancy.

## Case Presentation

**Figure 1:**
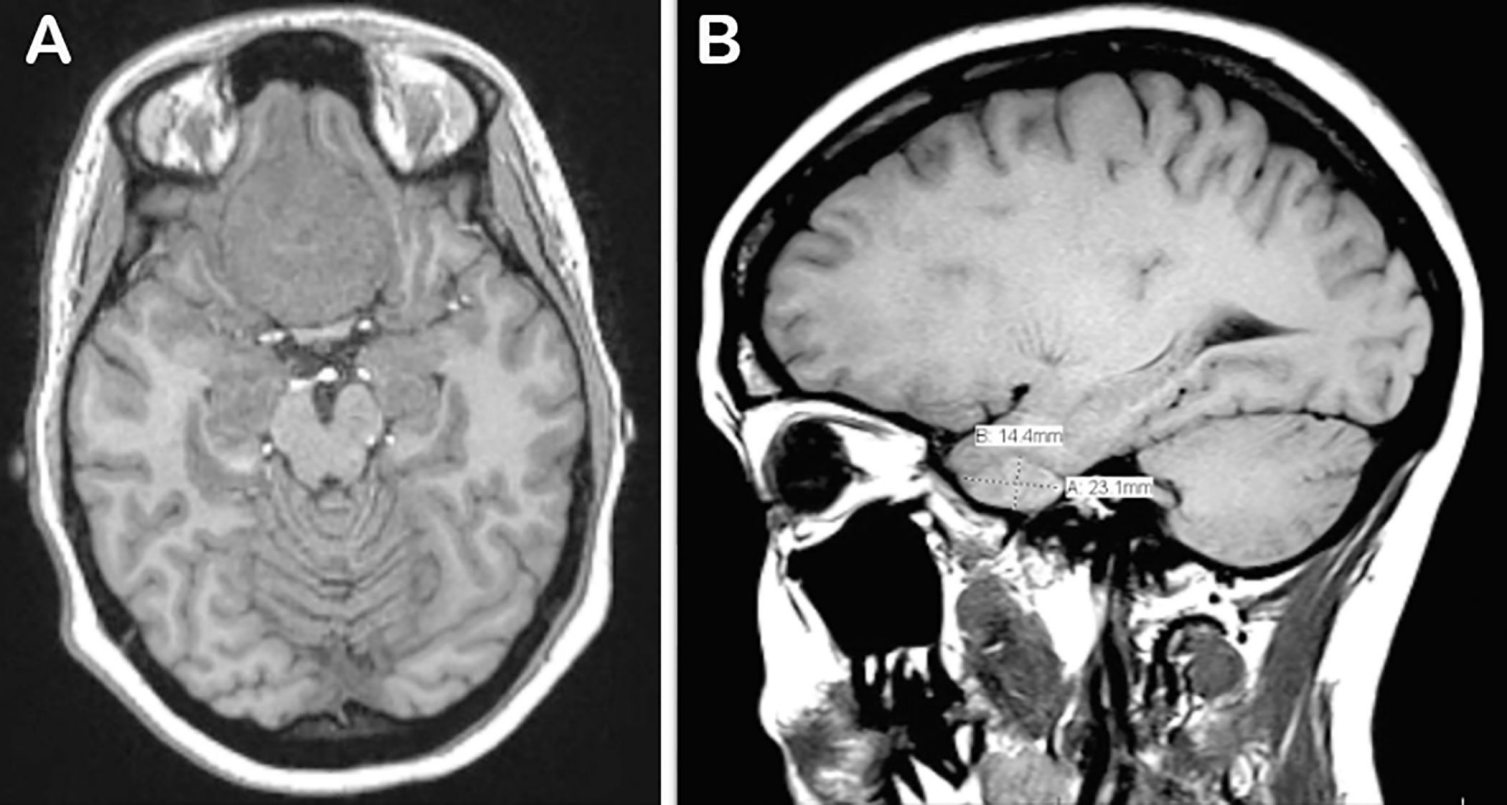
Pre-operative images. A) Axial T1 MRI revealed a large extra-axial meningioma within the anterior cranial fossa over the tuberculum sella with mass effect on optic chiasm and the prechiasmatic optic nerves. B) Sagittal T1 MRI revealed small extra-axial meningioma along right medial sphenoid wing.

**Figure 2:**
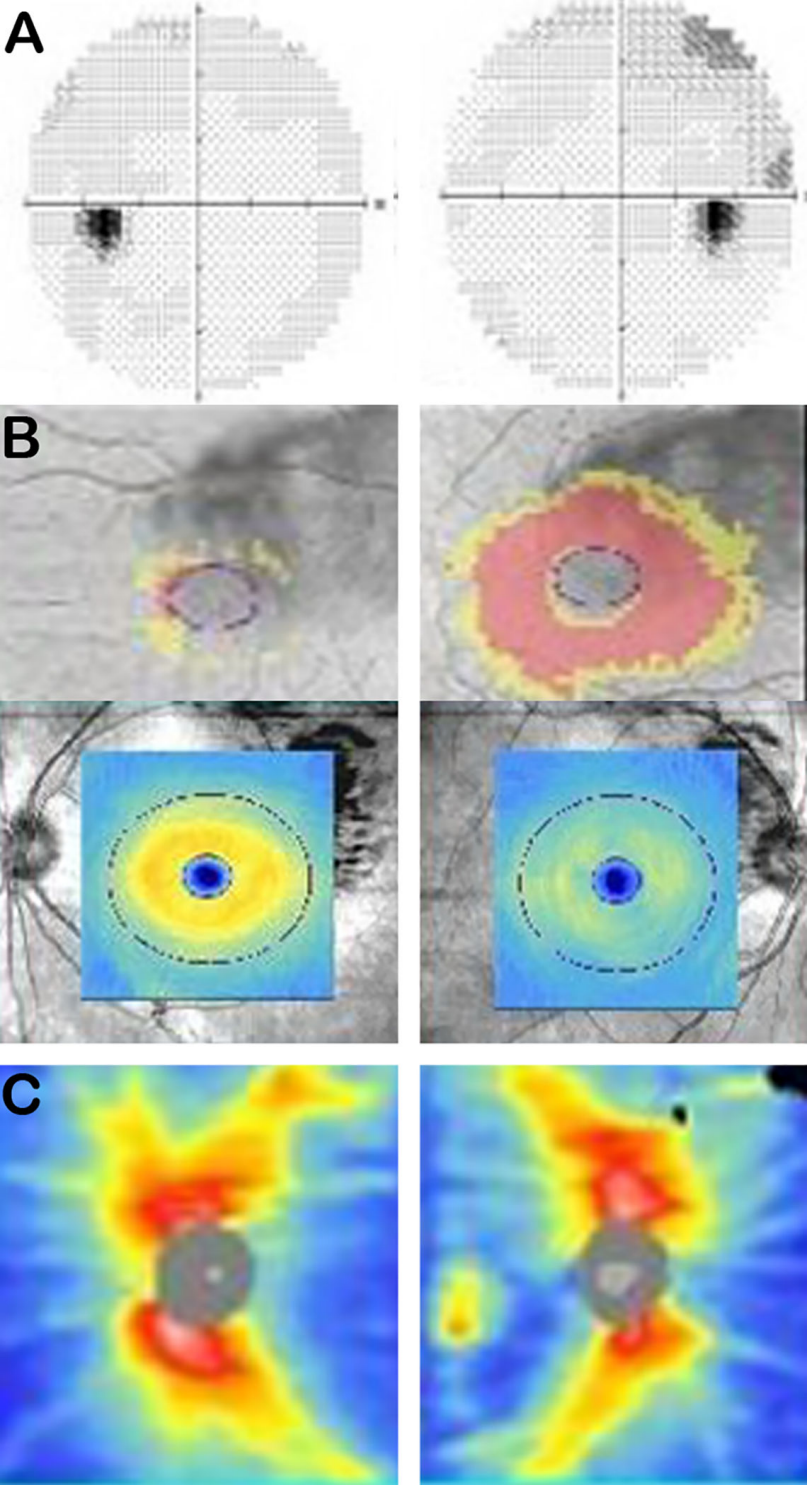
Post-operative images. A) Humphrey visual fields reveal overall preserved peripheral vision in both eyes. B) Retinal nerve fiber layer thickness was preserved in both eyes. C) Ganglion cell layer was thinned in right eye compared with left eye.

A 31-year-old primagravid female at 27 weeks gestation presented to the emergency room with a three-week history of progressive, painless bilateral blurred vision associated with intermittent headaches. Her past medical history was unremarkable. Notably, the patient had no complications with her current pregnancy, or a history of cancer or thromboembolic disease. On initial presentation, visual acuity was diminished to 20/200 in both eyes. There was no afferent pupillary defect or restriction of extraocular movements. She could only identify 4/15 color plates of the right eye and 5/15 of the left eye. Confrontation visual fields were full on the right but constricted on the left. No overt optic disc edema was observed. Given the history of pregnancy and progressive vision loss, magnetic resonance imaging (MRI) of the brain and orbits was obtained. Imaging revealed a large extra-axial mass with cystic degeneration centered within the anterior cranial fossa over the tuberculum sella extending to the level of the olfactory groove, measuring 4.5 cm × 4.0 cm × 2.5 cm in size, with mass effect compressing the optic chiasm and pre-chiasmatic optic nerves (Figure 1A). The lesion was isointense to gray matter on both T1 and T2 imaging, with areas of internal hemorrhage, calcification, and cystic degeneration. The mass abutted the pituitary gland but the gland itself was normal. An additional small extra-axial lesion was noted along the medial aspect of the right middle cranial fossa. Notably, the intraorbital optic nerves had normal signal. These findings were deemed most consistent with the presence of two intracranial meningiomas: a large tuberculum meningioma and a right medial sphenoid wing meningioma (Figure 1B). After discussing these results with the patient’s obstetrics and gynecology and neurosurgery teams, the decision was made to resect the tumors in order to preserve the patient’s vision. She underwent a right frontal temporal craniotomy with placement of a right frontal extraventricular drain with intrauterine monitoring. Intraoperatively, she was found to have a 12 cm tuberculum meningioma as well as a 3 cm sphenoid wing meningioma. Neurosurgery was able to achieve near total resection of the tumors with only a small residual amount of tumor left behind over the lateral aspect of the left optic nerve. Post-operatively, the patient’s vision improved significantly. Examination revealed 20/25 vision of both eyes with restoration of confrontation visual fields and color discrimination. Anterior and posterior segment examinations remained unchanged. Tests revealed intact peripheral visual fields and normal retinal nerve fiber layer thickness in both eyes (Figure 2A and 2B). There was, however, some residual thinning of the retinal ganglion cell layer of the right eye (Figure 2C). Final histopathological analysis revealed a grade 1 meningioma. A caesarean section was scheduled at 39 weeks with uncomplicated delivery of a female child. The patient will continue to be monitored closely for any visual deficits or recurrences of her tumors.

## Discussion

Due to variance in size and location, meningiomas can vary in clinical presentation. In most cases, they present as slow-growing tumors and are often asymptomatic. When located in the sphenoid wing, these tumors can cause unilateral visual field and visual acuity loss [4]. When located in the parasellar region, these tumors can cause bilateral visual loss, which can be exacerbated as the pituitary gland enlarges during pregnancy [5]. Reports of cranial nerve dysfunction and seizures are seen less frequently than visual complaints [6]. It is thought that the origin and growth of meningiomas may be related to female sex steroid production. Meningiomas have a higher incidence in females and 80% of benign meningiomas express progesterone receptors [3]. It is hypothesized that meningiomas grow more aggressively during pregnancy due to hormone induced cell proliferation secondary to elevated levels of progesterone, as well as increased hydration states [3]. Of note, the level of progesterone receptors has been noted to be inversely correlated with the histologic grade and mitotic index in meningiomas, suggesting that progesterone receptors may be more involved in the formation of benign rather than aggressive tumors [7]. Conversely, menopausal women may have a reduced risk of meningioma growth due to lower hormone levels [3,6]. In the majority of cases of meningiomas, observation is the traditional treatment approach. However, when neurologic symptoms arise or if the optic apparatus is involved, a more aggressive surgical approach is preferred in order to prevent permanent sequelae such as neurologic deficits or vision loss [8]. This approach is, however, complicated when the patient is pregnant as such surgery carries increased risk of morbidity and mortality to both mother and fetus [3]. Indications for urgent surgical intervention include parasellar location close to the optic apparatus and compression of the optic structures. The ideal timing of surgery depends on severity of damage to the optic structures, neurologic symptoms, maternal risk, and gestational age of the fetus [9]. Post-operative outcomes depend on the duration of symptoms, preoperative visual status and appearance of the optic disc, tumor size, adherence to vasculature, and peritumoral edema [8]. A study by Moscovici et al. [3] looked at 11 pregnant women with parasellar meningiomas involving the optic tract. Three of the patients diagnosed at 32 to 35 weeks gestation were observed and had spontaneous visual improvement following delivery. However, six of the patients with earlier diagnosis underwent surgical resection of their tumors. Of these patients, four underwent surgery at 20 to 23 weeks gestation with good visual outcomes and restoration of visual field deficits post-operatively; two patients who delayed surgery until after delivery suffered permanent unilateral blindness. Cohen-Gadol et al. [10] reviewed the neurosurgical treatment of 34 pregnant women with intracranial lesions. Among all, 56% underwent a neurosurgical procedure which included craniotomy with clipping and/or resection of lesions, stereotactic biopsies, or cerebrospinal fluid shunting procedures. Based on their experience, they found that surgery for intracranial lesions in pregnant lesions was overall well tolerated by both the mother and fetus. Interestingly, 9% of these patients successfully delivered by caesarean section prior to their neurosurgical procedure and 15% of them underwent therapeutic abortion preoperatively to allow for radiation therapy. When surgical intervention is not possible, the clinician needs to consider alternative treatment modalities such as external-beam radiation, partial excision followed by adjuvant radiotherapy, intensity-modulated radiation therapy, stereotactic radiotherapy or the use of chemotherapeutic agents or medications addressing newer molecular targets [7]. However, the use of these radiation or chemotherapeutic agents in pregnancy is difficult as they can threaten the life of the fetus. As such, termination of pregnancy or early delivery may be required to allow for complete treatment of the tumors [7]. Other considerations include the use of imaging modalities and administration of anesthesia in pregnant patients. Ongoing radiographic evaluation of intracranial tumors is necessary even in the pregnancy patient. Most studies find that the use of magnetic resonance imaging is safe during pregnancy; however, animal studies have shown intravenous gadolinium to be teratogenic at high and repeated doses. As such, it is recommended that gadolinium not be administered in pregnancy unless there is an essential clinical indication [11]. When performing non-obstetric surgery during pregnancy, consultation with anesthesiology is critical to determine optimal anesthesia management and post-operative analgesia. Maternal and fetal safety, teratogenicity of various anesthetic agents, and risk of preterm labor must all be considered [12]. From an ophthalmology standpoint, workup of a suspected meningioma should begin with a comprehensive history and exam. Visual acuity and visual field testing should be performed, but imaging is needed to confirm the diagnosis [3]. T2 weighted MRI is the preferred imaging modality [3,13]. Once the diagnosis is confirmed and the optic nerve apparatus is found to be compromised, vision is best preserved by attempting surgical resection as quickly as possible [3]. Earlier tumor resection can often result in improved visual outcomes [14]. It must also be noted that meningiomas are just one of the ocular pathologies that can cause worsening vision loss during pregnancy. A judicious workup and thorough differential diagnosis is warranted. Pituitary adenomas can cause compression of the chiasm and can enlarge over the course of pregnancy due to hormonal changes [15]. Graves’ disease may also worsen early in pregnancy, with approximately 25% of these cases involving ocular disturbances [16]. Diabetic retinopathy can accelerate during pregnancy; although most cases are mild, vision-threatening progression may occur more frequently when poorly controlled [17]. Lastly, hormonal changes in pregnancy may worsen the ocular pain and vision loss associated with uveal melanoma [18]. Vision loss in the pregnant patient is concerning and warrants a thorough and timely workup. If the presence of symptoms such as headache is accompanied by visual acuity and visual field loss, a meningioma must be considered as part of the differential diagnosis. If this diagnosis is confirmed by imaging studies, surgical resection should be considered to preserve vision. However, thorough planning and a team-based approach between obstetrics and gynecology, ophthalmology, neurosurgery and anesthesiology must be adopted in order to optimize outcomes for both the mother and fetus.
